# Aptamers as Innovative Tools for Prostate Cancer: Tumor Recognition and Biological Activity

**DOI:** 10.3390/biom16101412

**Published:** 2026-09-29

**Authors:** Tayanne Felippe Sassaro, Danielle Dias Pinto Ferreira, Aline dos Santos Moreira, Rayane da Silva Abreu, Natassia Silva de Araújo, Nina Carrossini Bastos, Priscila Valverde Fernandes, Wim Maurits Sylvain Degrave, Mariana Caldas Waghabi

**Affiliations:** 1 Laboratório de Genômica Aplicada e Bioinovações, Instituto Oswaldo Cruz, Fiocruz, Rio de Janeiro 21040-360, Brazil; tayannefelippe@gmail.com (T.F.S.); danielle.pinto@fiocruz.br (D.D.P.F.); amoreira@ioc.fiocruz.br (A.d.S.M.); rayane.abreu04@gmail.com (R.d.S.A.); natassiasaraujo@gmail.com (N.S.d.A.); wim.degrave@fiocruz.br (W.M.S.D.); 2 Divisão de Patologia (DIPAT), Instituto Nacional do Câncer (INCA), Rio de Janeiro 20220-400, Brazil; nina.bastos@inca.gov.br (N.C.B.); pvalverde@inca.gov.br (P.V.F.)

**Keywords:** prostate cancer, aptamers, diagnosis, therapy

## Abstract

Prostate cancer (PC) is the second most common malignancy among men worldwide and remains a leading cause of cancer-related mortality, highlighting the need for novel molecular tools for diagnosis and targeted therapy. In this study, five DNA aptamers (AptaP1–AptaP5) generated by Cell-SELEX against the LNCaP prostate cancer cell line were characterized for their structural features, tumor recognition, and biological activity. Secondary structure prediction and thermodynamic stability were evaluated in silico. Aptamer recognition was evaluated by flow cytometry in prostate cancer cell lines and by complementary aptafluorescence analyses in both cell lines and a prostate tissue microarray (TMA), while functional effects were evaluated by assessment of metabolic activity and proliferation assays. The selected aptamers preferentially recognized LNCaP and DU145 cells over RWPE-1, whereas no tumor-selective binding was observed for PC3 cells. Aptafluorescence analyses in the prostate TMA demonstrated differential tissue recognition, with signals detected across both primary and metastatic prostate cancer specimens. Moreover, all aptamers significantly reduced metabolic activity and proliferation in prostate cancer cells after 48 h of treatment. Collectively, these findings characterized novel DNA aptamers with promising potential as tools for prostate cancer detection and provide a basis for further investigation of their binding properties and biological effects in clinically relevant models.

## 1. Introduction

Prostate cancer (PC) is the second most common malignancy among men worldwide, excluding non-melanoma skin cancers [1]. Despite substantial advances in diagnosis and treatment, PC remains one of the leading causes of cancer-related death in men. Its marked molecular and clinical heterogeneity poses major challenges for early diagnosis, prognostic stratification, and the development of broadly applicable targeted therapies.

Current clinical evaluation of prostate cancer relies on complementary approaches with distinct diagnostic and clinical roles. Serum prostate-specific antigen (PSA) is widely used for initial assessment and risk stratification, but it is organ-specific rather than cancer-specific, as elevated levels may also occur in benign prostatic hyperplasia, prostatitis, and other non-malignant conditions [2]. Definitive diagnosis, however, requires histopathological evaluation of prostate tissue, typically obtained by biopsy. Molecular imaging with prostate-specific membrane antigen (PSMA)-targeted radiotracers has further expanded the clinical assessment of prostate cancer, particularly for disease localization, staging, and detection of recurrent or metastatic disease [3]. Nevertheless, PSMA expression is heterogeneous across prostate cancer, with interpatient, intratumoral, and temporal variation, as well as changes associated with tumor lineage and disease progression [4]. Reduced PSMA expression has been reported in subsets of advanced prostate cancers, including tumors exhibiting neuroendocrine differentiation, which may limit the sensitivity or applicability of PSMA-directed imaging and therapeutic approaches in selected cases [4,5]. These considerations support continued investigation of alternative molecular recognition agents that may complement existing diagnostic and imaging strategies. In this context, aptamers offer a versatile platform for molecular recognition and may have potential applications in the detection of prostate cancer cells. Aptamers have emerged as promising molecular recognition agents with considerable potential to overcome these limitations. Aptamers are short single-stranded DNA or RNA oligonucleotides that fold into defined three-dimensional conformations, enabling high-affinity and highly specific binding to molecular targets [6]. Compared with monoclonal antibodies, aptamers offer several practical advantages, including chemical synthesis with minimal batch-to-batch variation, low immunogenicity, efficient tissue penetration due to their small size, and straightforward chemical modification for conjugation with imaging probes or therapeutic cargos [7,8,9]. These characteristics make aptamers attractive candidates for theranostic applications by integrating diagnosis and targeted therapy within a single molecular platform.

Several aptamers have been developed for prostate cancer and have demonstrated promising applications in molecular imaging, biosensing, targeted drug delivery, and cancer therapy [10,11,12]. Among the available selection strategies, Cell-SELEX has emerged as a powerful approach for generating aptamers against intact living cells, enabling the identification of ligands based on differential cellular recognition without prior knowledge of the target molecule [13]. This strategy is particularly attractive for complex and heterogeneous diseases such as prostate cancer, in which the molecular targets responsible for aptamer binding may not be known a priori.

Using this strategy, our group previously selected five DNA aptamers [14] against the human prostate cancer cell line LNCaP, an androgen-sensitive, PSMA-positive model, using the non-tumoral prostate epithelial cell line RWPE-1 as a counter-selection control. Although these aptamers were successfully enriched for selective recognition of tumor cells, their structural features, biological activity, and ability to recognize distinct prostate cancer phenotypes have not yet been comprehensively investigated. To address this, we evaluated the aptamers using prostate cancer models representing different molecular and biological characteristics. Besides LNCaP, we used DU145 and PC3, two androgen-independent cell lines exhibiting low and absent PSMA expression, respectively, providing practical in vitro models of PSMA-heterogeneous prostate cancer.

In the present study, we characterize these aptamers in depth, evaluating their predicted secondary and three-dimensional structures, binding profiles, and biological effects across these distinct prostate cancer cell lines, as well as their recognition of primary and metastatic patient-derived prostate tissues. We demonstrate that selected aptamers exhibit distinct binding patterns across heterogeneous prostate cancer models, impair tumor cell metabolic activity and proliferation, and selectively recognize malignant prostate tissues, supporting their further development as molecular tools for prostate cancer diagnosis and targeted therapeutic applications.

## 2. Materials and Methods

### 2.1. Cell Lines and Culture Conditions

The metastatic prostate cancer cell lines LNCaP, PC3, and DU145, as well as the non-tumoral prostate epithelial cell line RWPE-1, were used in this study. LNCaP cells were obtained from the INCA Cell Line Collection and kindly provided by Etel Gimba. RWPE-1, PC3, and DU145 cells were acquired from the Rio de Janeiro Cell Bank (BCRJ). Cell line authenticity was subsequently confirmed by BCRJ and the DNA Fragment and Microsatellite Analysis Platform (RPT01D) at Fiocruz (https://plataformas.fiocruz.br/, accessed on 3 November 2022).

LNCaP, PC3, and DU145 cells were cultured in RPMI-1640 medium (Sigma-Aldrich, St. Louis, MO, USA) supplemented with 2 g/L sodium bicarbonate, 25 mM HEPES, 10% fetal bovine serum (FBS), and 1% penicillin–streptomycin solution (Sigma-Aldrich). RWPE-1 cells were maintained in Keratinocyte Serum-Free Medium (Thermo Fisher Scientific, Waltham, MA, USA) supplemented with 50 mg/L bovine pituitary extract (BPE), 5.0 µg/L recombinant human epidermal growth factor (rhEGF), and 1% penicillin–streptomycin solution (Sigma-Aldrich). All cell cultures were maintained at 37 °C in a humidified incubator with an atmosphere containing 5% CO_2_.

### 2.2. Aptamers Previously Selected Against LNCaP Cells

The DNA aptamers evaluated in this study were previously selected by our group against the prostate cancer cell line LNCaP using the Cell-SELEX methodology. After 12 rounds of selection and amplification, enriched aptamer sequences were identified by next-generation sequencing, and the five most enriched sequences (AptaP1–AptaP5) were selected for further characterization in the present study. Detailed information regarding the Cell-SELEX procedure and sequence selection has been previously described [14]. Each aptamer is approximately 76 nucleotides in length without terminal modifications. The sequences are currently under patent protection (N°: PCT/BR2022/050356) and cannot be disclosed. Before use, the aptamers were diluted in Binding Buffer (500 mL DPBS supplemented with 4.5 g glucose, 1 g BSA, and 5 mL of 1 M MgCl_2_), heated at 95 °C for 10 min, and subsequently cooled on ice to allow individual aptamer folding.

### 2.3. Secondary and Tertiary Structure Prediction of Aptamers

The secondary structures of the aptamers were initially predicted using the UNAfold Web Server with the mFold extension (http://mFold.rna.albany.edu/?=mFold; accessed on 10 December 2022). To independently assess the predicted structures, the aptamer sequences were also analyzed using the NUPACK web server (http://www.nupack.org; accessed on 10 December 2022). Both platforms generated predictions of the secondary structure of aptamers in dot-bracket notation and provided the corresponding Gibbs free energy (ΔG) values.

Because computational approaches for predicting the three-dimensional structures of ssDNA aptamers remain relatively limited, a previously described strategy [15,16] was adopted based on the methodology proposed by [17]. Briefly, the DNA sequences were temporarily converted into RNA sequences by replacing thymine with uracil. The resulting RNA sequences, together with their corresponding secondary structures in dot-bracket notation, were submitted to the RNAComposer server (https://rnacomposer.cs.put.poznan.pl/; accessed on 20 December 2022) for tertiary structure prediction.

The predicted RNA structures were subsequently converted back to their DNA counterparts using the x3DNA server (http://web.x3dna.org/index.php/mutation; accessed on 21 December 2022). During this step, uracil residues were replaced with thymine and ribose sugars were converted to deoxyribose, generating the corresponding three-dimensional DNA aptamer structures. The resulting PDB files were further subjected to geometric refinement and energy minimization using PHENIX software, version 1.19.2-4158 [18].

To assess the structural consistency of the independent secondary-structure predictions, the three-dimensional models generated from the mFold- and NUPACK-derived secondary structures were structurally aligned for each of the selected aptamers using PyMOL, version 3.0.3. Structural similarity between the corresponding models was quantified by calculating the RMSD using the PyMOL align command. RMSD represents the root-mean-square deviation between equivalent atom pairs following structural superposition. An RMSD value of zero corresponds to identical structures after superposition; therefore, values closer to zero indicate greater structural similarity between the corresponding mFold- and NUPACK-derived models, whereas increasing RMSD values indicate progressively greater structural differences. Here, RMSD was used to evaluate the degree of agreement between the independent computational predictions.

### 2.4. Evaluation of Specificity of Aptamers by Flow Cytometry

Following identification of the aptamer sequences enriched during selection against LNCaP cells, the five most abundant sequences obtained in the final selection round were selected for evaluation of their binding specificity. All cells were harvested using 0.25% Trypsin/EDTA (Sigma-Aldrich) and counted with an automated cell counter (Countess II, Thermo Fisher Scientific, Waltham, MA, USA). For each assay, 5 × 10^5^ cells were incubated with FAM-conjugated aptamers at a final concentration of 200 or 400 nM for 1 h in 5% CO_2_ at 37 °C. These concentrations were used for comparison of binding, and Kd values were not determined.

Library-Binding Buffer, composed of DPBS supplemented with 4.5 g glucose, 1 g BSA, and 5 mL of 1 M MgCl_2_, was used as a negative control, together with the initial random-sequence library at a concentration of 400 nM. The Wy-5a aptamer, previously reported to recognize prostate cancer cell lines, was included as a reference aptamer at 200 or 400 nM.

Following incubation, the cells were centrifuged to remove unbound aptamers and subsequently washed with Washing Buffer. Samples were analyzed using a Cytoflex flow cytometer (Beckman Coulter, Brea, CA, USA) at the Flow Cytometry platform of Oswaldo Cruz Foundation. Data acquisition and analysis were carried out using CytExpert software (v2.5; Beckman Coulter, Brea, CA, USA).

Flow-cytometry gating was performed according to standard procedures. Briefly, cellular debris was excluded by identifying the main cell population based on Forward Scatter Area (FSC-A) versus Side Scatter Area (SSC-A), followed by exclusion of doublets and cell aggregates using FSC-Height (FSC-H) versus FSC-A plots. Binding (%) was determined within the resulting intact singlet cell population based on fluorescence above the background defined by an unselected randomized oligonucleotide library labeled with the same fluorophore, which was used as the negative control.

Regarding the number of events and biological replicates, approximately 10,000 singlet events were acquired per sample for flow-cytometry analysis.

Statistical analysis was performed using a one-way ANOVA with Tukey’s multiple-comparisons test to compare the binding of each aptamer with each negative control and between the two concentrations within the same treatment.

For the aptamer binding assay comparing tumor and non-tumor cells, a two-way ANOVA with Sidak’s multiple-comparisons test was used to compare the aptamer binding between tumor and non-tumor cells.

### 2.5. Evaluation of Specificity of Aptamers by Aptafluorescence

Prostate tumor cell lines LNCaP, PC3 and DU145 and the non- tumor cell line RWPE were seeded at a density of 5 × 10^5^ cells per chamber in Labtek chamber slides (Thermo Scientific™). Prior to aptamer addition, the cells were incubated with Binding Buffer consisting of 500 mL DPBS supplemented with 4.5 g glucose, 1 g BSA, and 5 mL of 1 M MgCl_2_. The cells were then incubated with FAM-conjugated aptamers for 1 h in 5% CO_2_ at 37 °C. Following incubation, the cells were washed in PBS and fixed with 4% paraformaldehyde for 5 min. Nuclear staining was subsequently performed by incubating the cells with DAPI (1:5000) for 10 min, followed by an additional PBS wash. The slides were mounted using DABCO (Sigma-Aldrich), and fluorescence images were acquired using a Nikon Eclipse microscope (Nikon Instruments, Tokyo, Japan) with a 20× objective and NIS-Elements software (v5.42; Nikon Instruments, Tokyo, Japan).

### 2.6. Assessment of Cellular Metabolic Activity

To assess the metabolic activity of tumor cells following aptamer treatment, a resazurin-based redox assay was performed using the PrestoBlue Cell Viability Reagent (Thermo Fisher Scientific). For this assay, 5 × 10^4^ cells from prostate metastatic cell lines (LNCaP, PC3 and DU-145) were seeded in 96-well plates. After 24 h of cell seeding, metabolic activity was measured immediately before aptamer treatment and defined as the 0 h time point. Cells were then treated with 400 nM of each individual aptamer, and metabolic activity was evaluated at 24 and 48 h post-treatment using separate wells for each time point. All measurements were performed as independent endpoint assays. At each time point, 10 μL of PrestoBlue reagent was added to each well, and the plates were incubated for 1 h at 37 °C in a CO_2_ atmosphere. Fluorescence readings were performed using the Varioskan LUX multimode microplate reader (Thermo Fisher Scientific, Waltham, MA, USA) at excitation and emission wavelengths of 560 nm and 590 nm, respectively. Three independent biological assays were performed in technical triplicates.

For the evaluation of the effect of the aptamers at each time point, a two-way ANOVA was performed with treatment condition and time as factors. Dunnett’s multiple-comparisons test was subsequently applied using the untreated negative control as the reference condition. Comparisons were restricted to treatment-versus-control comparisons at 24 and 48 h. The positive control was included as an experimental control for assay validation.

For the evaluation of the effect of the aptamers over time, a two-way ANOVA was performed with Tukey’s multiple-comparisons test to compare the three time points within each treatment condition separately (0 vs. 24 h, 24 vs. 48 h, and 0 vs. 48 h).

### 2.7. Cell Proliferation Assay

The effects of the selected aptamers on the proliferation of the prostate cancer cell lines LNCaP, PC3, and DU145 were evaluated using the CyQUANT Cell Proliferation Assay (Thermo Fisher Scientific).

Briefly, 5 × 10^4^ cells were seeded into black polystyrene 96-well plates (Greiner Bio-One, Kremsmünster, Austria) and maintained at 37 °C in a humidified atmosphere containing 5% CO_2_. Upon reaching approximately 90% confluence, cells were incubated individually with each selected aptamer at a final concentration of 400 nM for 48 h.

At each experimental time point, the CyQUANT reagent was added to the wells and incubated for 30 min according to the manufacturer’s instructions. Fluorescence was measured using a FlexStation 3 microplate reader (Molecular Devices, San Jose, CA, USA) available at the Bioassays Platform of the Oswaldo Cruz Institute, using excitation and emission wavelengths of 480 and 520 nm, respectively. The fluorescence value obtained from cells maintained in culture medium without aptamer treatment was defined as 100% proliferation. The proliferation index for each treatment condition was calculated by expressing its fluorescence value relative to the untreated control and reported as a percentage of the control.

Statistical analysis was performed using a one-way analysis of variance (ANOVA), followed by Dunnett’s multiple-comparisons test, with the untreated control condition used as the reference group.

### 2.8. Validation of Aptamer Recognition in Prostate Cancer Samples Using Tissue Microarray (TMA)

The binding of the aptamers to clinical prostate cancer specimens was assessed using a tissue microarray (TMA) slide obtained from US Biomax Inc. (Rockville, MD, USA; Ref. PR8011a). The slide contained 30 cores of adenocarcinoma, 2 cores of small acinar carcinoma, 2 cores of metastatic adenocarcinoma, 26 cores of hyperplastic prostate tissue, 6 cores of chronic inflammation, 6 cores of adjacent normal prostate tissue and 8 cores of prostate tissue. Prior to processing, the slides were incubated at 60 °C for 2 h. Paraffin removal was performed by three consecutive washes in xylol for 5 min each, followed by rehydration through a graded ethanol series (100%, 95%, 80%, and 70%), with each step lasting 5 min. The tissue sections were subsequently immersed in water for 5 min.

Antigen retrieval was performed by placing the slides in a steamer containing Citrate pH 6.0/Tris-EDTA pH 9.0/Trilogy™ (Cell Marque, Rocklin, CA, USA) solution for 30 min. The sections were then washed three times with TBS for 5 min each and incubated with Novolink™ Protein Block (Leica Biosystems, Deer Park, IL, USA) for 5 min to minimize nonspecific binding. The slides were subsequently incubated with 400 nM Cy5-conjugated AptaP1, AptaP2, AptaP3, AptaP4, or AptaP5 for 1 h at room temperature.

Following aptamer incubation, the sections were washed with TBS and counterstained with DAPI (1:5000) for 10 min. After an additional TBS wash, the slides were mounted using ProLong™ Gold Antifade reagent (Thermo Fisher Scientific). The mounting medium was allowed to polymerize for 24 h, after which fluorescence images were acquired using a Nikon Eclipse microscope and Nikon Instruments imaging software.

### 2.9. Statistical Analysis

Statistical analyses were performed according to the experimental design of each assay. Data are presented as mean ± standard deviation (SD) from at least n = 3 independent experiments. All statistical tests were two-sided, and *p* < 0.05 was considered statistically significant.

## 3. Results

### 3.1. Prediction and Analysis of Structures of Selected Aptamers for LNCaP Cells

As an initial step, we predicted the secondary structures of the selected aptamers using the ssDNA sequences of the five most frequent aptamers identified in the final round of selection for LNCaP cells. Secondary structure predictions were performed using the NUPACK (http://www.nupack.org) and mfold (http://mfold.rna.albany.edu/?=mfold) web servers, both of which rely on free energy minimization techniques. In mfold, all possible secondary structures are generated based on Watson–Crick base pairing, and the most thermodynamically stable structures are selected. This analysis also provides the minimum free energy (ΔG) associated with each aptamer fold. Sequences with lower ΔG values, indicative of more stable structures, were prioritized, along with those exhibiting consistent structural predictions across both NUPACK and mfold.

Among the five selected aptamers for LNCaP cells, AptaP1 and AptaP4 displayed highly similar predicted structures across both applications. These aptamers exhibited extensive internal base pairing and a high prevalence of hairpin loops (Figure 1). The structures associated with the lowest free energy (ΔG) were selected for further analysis, as these represent the most likely spontaneous and stable conformations. Figure 1 summarizes the ΔG values obtained for the predicted structures of each aptamer using both applications. Notably, all ΔG values for the secondary structures were negative, confirming that the predicted conformations are thermodynamically favorable and potentially spontaneous (Figure 1).

Previous studies from our group [15,16] refined a computational method for predicting ssDNA 3D structures based on the methodology developed by [17]. Following this approach, we first input the consensus secondary structures, predicted by both mfold and NUPACK, into the RNA Composer platform. This step generates 3D RNA structures derived from the 2D ssDNA sequences. Subsequently, using the 3DNA server, the 3D RNA structures were converted into 3D ssDNA by automatically replacing ribose sugars with deoxyribose and uracil bases with thymine. Representative 3D structures of each aptamer (AptaP1–AptaP5), predicted from their respective secondary structure data, are presented in Figure 1.

To assess the consistency between the structural predictions obtained using the two computational approaches, the corresponding three-dimensional models were structurally aligned, and their RMSD values were calculated. AptaP2, AptaP4, and AptaP5 showed low RMSD values (0.12, 0.46, and 0.23, respectively), indicating a high degree of structural similarity between the models generated from mFold and NUPACK predictions. AptaP1 showed an intermediate RMSD value of 1.38, suggesting greater structural variation between the two predicted models. In contrast, AptaP3 exhibited the highest RMSD value (6.11), indicating substantial structural divergence between the models generated by the two prediction approaches (Figure 1).

### 3.2. Binding Profile of Selected Aptamers Across Prostate Cell Lines

Since the selected aptamers were isolated by Cell-SELEX using LNCaP cells as the target, we next investigated whether they also recognized other prostate cancer cell lines at two different aptamer concentrations. FAM-conjugated aptamers were evaluated by flow cytometry in LNCaP, DU145, and PC3 cells at 200 and 400 nM. All the five selected aptamers exhibited measurable binding to the three prostate cancer cell lines, although binding intensity varied among aptamers and across cell lines. Each histogram is presented in Supplementary Figures (Figures S1–S4). Across the aptamer panel, binding was highest in LNCaP cells, intermediate in DU145, and lowest in PC3 cells (Figure 2). The binding results obtained at the two concentrations provided an initial assessment of concentration-dependent differences in aptamer recognition. Based on these initial experiments, 400 nM was selected for the subsequent functional and characterization assays.

To benchmark the selected aptamers against a previously reported prostate cancer-targeting aptamer, the same assay was performed with Wy-5a under identical experimental conditions. Originally selected and characterized using PC3 cells [10], Wy-5a exhibited the highest binding to PC3 cells among all selected aptamers (Figure 2).

### 3.3. Tumor Selectivity of the Selected Aptamers

To evaluate tumor selectivity, the binding of each aptamer to prostate cancer cell lines was compared with its binding to the non-tumorigenic prostate epithelial cell line RWPE-1. Except for AptaP1, all selected aptamers exhibited significantly higher binding to LNCaP and DU145 cells than to RWPE-1. Among the aptamers evaluated in LNCaP cells, AptaP4 showed the highest binding, recognizing 44.53% of the cells, corresponding to a 5.2-fold increase compared with RWPE-1 (*p* < 0.0001). In DU145 cells, AptaP5 exhibited the highest binding, recognizing 32.7% of the cells, corresponding to a 3.9-fold increase compared with RWPE-1 (*p* < 0.0001). In contrast, none of the selected aptamers showed significantly higher binding to PC3 cells than to RWPE-1. Notably, AptaP1 bound significantly less to PC3 than to RWPE-1 cells.

Wy-5a exhibited a similar selectivity profile, showing significantly higher binding to LNCaP and DU145 than to RWPE-1, while failing to discriminate PC3 cells from the non-tumorigenic prostate cell line (Figure 3).

### 3.4. Aptafluorescence Profiling of Aptamers Across Prostate Cell Lines

To further characterize aptamer binding by fluorescence microscopy, FAM-conjugated aptamers (AptaP1–AptaP5) and the aptamer Wy-5a were incubated with LNCaP, DU145, PC3, and RWPE-1 cells, followed by qualitative fluorescence imaging (Figure 4). All five selected aptamers and Wy-5a, produced detectable fluorescence in LNCaP cells. In DU145 cells, fluorescence was observed only for AptaP2, AptaP4, and AptaP5, whereas AptaP1, AptaP3, and Wy-5a showed no detectable signal. No fluorescence was detected in PC3 or RWPE-1 cells with any of the evaluated aptamers.

### 3.5. Aptamer Specificity in Prostate Cancer Tissue Samples

We next investigated whether the selected aptamers could also recognize human prostate tumor tissues. For this purpose, Cy5-conjugated aptamers were evaluated by fluorescence microscopy using a prostate tissue microarray containing normal prostate tissue, hyperplasia, adenocarcinoma Grade Group 1 (GG1), and metastatic adenocarcinoma specimens.

Microscopic analysis revealed stronger aptamer staining in malignant tissues compared with non-neoplastic samples. In metastatic adenocarcinoma specimens, AptaP4 and AptaP5 exhibited the strongest fluorescence signals, whereas AptaP3, AptaP4, and AptaP5 showed the most intense staining in GG1 adenocarcinoma samples. Conversely, hyperplastic and normal prostate tissues displayed markedly weaker fluorescence for all evaluated aptamers (Figure 5).

### 3.6. Functional Evaluation of Aptamers in Prostate Cancer Cell Lines

To evaluate the effects of the selected aptamers on prostate cancer cells, metabolic activity was assessed using the resazurin reduction assay. LNCaP, PC3 and DU145 cells were incubated with 400 nM of AptaP1–AptaP5, while RPMI medium and Triton X-100 (0.01%) served as negative and positive controls, respectively.

At 24 h, none of the selected aptamers significantly affected metabolic activity compared with the medium control in any of the three cell lines. However, after 48 h, all five aptamers showed significantly reduced metabolic activity relative to the medium control in LNCaP, PC3, and DU145 cells (Figure 6). AptaP1 produced the greatest reduction in LNCaP and PC3 cells, decreasing metabolic activity by 46% and 55%, respectively (*p* < 0.0001 for both), whereas AptaP5 showed the strongest effect in DU145 cells, reducing metabolic activity by 41.8% (*p* < 0.0001).

Similarly, the aptamer Wy-5a significantly reduced cell metabolic activity compared with the medium control after 48 h in all three prostate cancer cell lines (Figure 6).

To determine whether aptamer treatment influenced the temporal evolution of cellular metabolic activity, fluorescence values obtained at 0, 24, and 48 h were compared within each treatment group. As expected, medium-treated cells exhibited a progressive increase in metabolic activity over time in all three prostate cancer cell lines, consistent with continued cell proliferation. In contrast, most aptamer-treated groups remained largely stable throughout the 48 h incubation period, with no significant changes across the evaluated time points (Figure 7).

In LNCaP cells, AptaP1, AptaP2, and AptaP5 showed no significant differences between any of the evaluated time points. AptaP3 and AptaP4 also showed no significant differences between consecutive time points (0–24 h and 24–48 h); however, both exhibited a significant increase of more than 70% at 48 h compared with baseline (*p* = 0.0300 and *p* = 0.0186, respectively) (Figure 7a).

In PC3 cells, none of the evaluated aptamers exhibited significant changes in metabolic activity across any of the analyzed time intervals (Figure 7b).

In DU145 cells, AptaP4 was the only aptamer showing significant temporal changes, with metabolic activity increasing 1.7-fold from 24 to 48 h (*p* = 0.0364) and 2.0-fold relative to baseline (*p* = 0.0126). No other within-group comparisons reached statistical significance (Figure 7c).Wy-5a exhibited a similar temporal profile, although significant increases were observed in LNCaP and DU145 cells. In LNCaP cells, metabolic activity at 48 h was 1.9-fold higher than at baseline (*p* = 0.0089) and 1.65-fold higher than at 24 h (*p* = 0.0287), whereas no significant difference was observed between 0 and 24 h (Figure 7a). In PC3 cells, no significant changes were detected across the evaluated time points (Figure 7b). In DU145 cells, metabolic activity at 48 h was 1.9-fold higher than at baseline (*p* = 0.0365), with no significant differences between consecutive time points (Figure 7c).

To determine whether the effects observed in metabolic activity were associated with impaired cell proliferation, proliferation was assessed after 48 h of aptamer treatment using the CyQUANT assay.

In LNCaP cells, all evaluated aptamers significantly reduced cell proliferation compared with the medium control, although the magnitude of the inhibitory effect varied among aptamers. Overall, the mean proliferation index was 50.73% (Figure 8a). The strongest inhibitory effect was observed for AptaP4, which reduced proliferation by 60% compared with the control (*p* = 0.0198). A more pronounced antiproliferative effect was observed in PC3 cells, in which all aptamers markedly reduced proliferation, resulting in a mean proliferation index of 12% (Figure 8b). In DU145 cells, all aptamers also significantly decreased proliferation, although the inhibitory effect was less pronounced than that observed in PC3 cells, with a mean proliferation index of 26.53% (Figure 8c). Notably, AptaP3 showed the strongest antiproliferative effect in both PC3 and DU145 cells, reducing proliferation by more than 90% (*p* < 0.0001) and 86% (*p* = 0.0006), respectively. For the reference aptamer Wy-5a, proliferation indexes were 20.67% in PC3 cells (*p* = 0.0001) and 38.00% in DU145 cells (*p* = 0.0004), while no significant effect was observed in LNCaP cells (*p* = 0.1729) (Figure 8). Overall, these findings demonstrate that all selected aptamers exert antiproliferative activity in metastatic prostate cancer cells, with the strongest effect observed in the PC3 cell line.

Together, these findings demonstrate that the tumor-selective binding observed in prostate cancer cell lines is preserved in human prostate tissue specimens, providing further evidence that the selected aptamers preferentially recognize malignant prostate tissue. Moreover, their ability to reduce cancer cell metabolic activity and proliferation highlights an additional functional effect beyond tumor cell recognition. Collectively, these findings support the potential of the selected aptamers as promising candidates for prostate cancer-targeted applications and warrant further investigation into their molecular targets and mechanisms of action.

## 4. Discussion

The present study describes the identification and characterization of five novel DNA aptamers targeting prostate cancer cells through Cell-SELEX. Beyond their successful selection, these aptamers demonstrated selective recognition of prostate cancer cell lines, differential binding to patient-derived prostate tumor tissues, and measurable inhibitory effects on cellular metabolic activity and proliferation. Collectively, these findings support the potential of this aptamer panel as a foundation for future diagnostic and therapeutic applications while also highlighting the value of using multiple complementary aptamers to address the molecular heterogeneity of prostate cancer.

Because aptamer function depends on its three-dimensional conformation, structural characterization represents an important step in the evaluation of candidate aptamers. Secondary structure predictions generated independently using mFold and NUPACK produced negative free-energy (ΔG) values for the predicted conformations of all five aptamers. These ΔG values were considered thermodynamic descriptors of the predicted structures and were not interpreted as evidence of biological or structural stability.

Although computational structural modeling cannot establish binding affinity, target identity, or biological activity, comparison of independently predicted structures provides a useful approach for evaluating the consistency of the predicted conformations [19,20]. The RMSD analysis was used to assess the structural agreement between the models generated from mFold and NUPACK, with lower values indicating greater structural similarity between the corresponding predictions. Thus, the observed differences in RMSD among the aptamers indicate varying degrees of agreement between the two computational approaches. These findings provide a useful framework for future characterization of the structural features of these aptamers.

Flow cytometry demonstrated that all five aptamers recognized the three prostate cancer cell lines evaluated, with the highest binding observed in LNCaP cells. This finding was expected, as LNCaP was the cell line originally used during aptamer selection. Importantly, the selected aptamers also recognized DU145 and PC3 cells, indicating that their binding is not restricted to the cell line used for selection. The differences in binding intensity observed among the prostate cancer cell lines may reflect variation in the presence, abundance, or accessibility of the molecular targets recognized by the aptamers across different cellular contexts. However, because the molecular targets have not yet been identified, the basis for these differences cannot be established from the present data.

Comparison with the non-tumoral RWPE-1 control provided a more informative assessment of tumor selectivity. AptaP2–AptaP5 exhibited significantly greater binding than RWPE-1 in both LNCaP and DU145 cells, whereas none of the aptamers demonstrated tumor-selective binding in PC3 cells. Fluorescence microscopy provided qualitative observations consistent with these findings, with fluorescence detected for all five aptamers in LNCaP cells, whereas only AptaP2, AptaP4, and AptaP5 produced detectable signals in DU145 cells. No detectable fluorescence was observed in PC3 or RWPE-1 cells. Although fluorescence microscopy is not directly quantitative, its agreement with the flow cytometry data strengthens the evidence for preferential recognition of LNCaP and DU145 cells. Together, these findings reinforce the importance of evaluating candidate aptamers across multiple experimental systems rather than relying on a single cell line. This is consistent with the findings reported by Kelly et al. [21], who demonstrated that several previously reported cell-targeting aptamers failed to reproduce their originally described specificity when assessed under standardized experimental conditions.

Several aptamers targeting prostate cancer have been described over the past two decades, reflecting the growing interest in aptamer-based strategies for cancer diagnosis and therapy [22,23]. Among them, the best-characterized example is the RNA aptamer A10, which recognizes PSMA and has been extensively investigated for targeted drug delivery, molecular imaging, and therapeutic applications [24,25,26]. More recently, the RNA aptamers D4 and R4 demonstrated promising performance for detecting circulating tumor cells in patients with prostate cancer [27]. Despite these advances, many currently available prostate cancer aptamers rely on PSMA as their principal molecular target [23,28]. Although PSMA is highly expressed in many prostate tumors, its expression is heterogeneous and is frequently reduced or absent in androgen-independent models such as DU145 and PC3 [29]. In the present study, four of the five DNA aptamers exhibited significantly greater binding to DU145 cells than to RWPE-1 cells, indicating that their recognition is not restricted to the androgen-sensitive phenotype used during selection. Although the molecular targets remain unknown and the contribution of PSMA to aptamer binding has not yet been determined, these findings demonstrate that the selected aptamers can recognize distinct prostate cancer phenotypes, including models with high or low PSMA expression, potentially broadening the spectrum of tumors that could be targeted. An additional advantage of the present candidates is that they are DNA aptamers, which generally exhibit greater chemical stability, lower production costs, and simpler chemical synthesis than RNA aptamers, features that may facilitate future translational development [30].

The aptamer Wy-5a provided an additional point of comparison for evaluating the binding profiles of the newly identified candidates. Originally selected and characterized using PC3 cells, Wy-5a has been reported to exhibit trypsin-sensitive binding, with reduced binding following trypsin treatment [10]. In the present study, all cell lines were processed under standardized conditions using trypsin for cell dissociation, and Wy-5a exhibited lower binding to PC3 cells than previously reported. Interestingly, the newly characterized aptamers also generally showed different binding to PC3 compared with the other prostate cancer cell lines evaluated. Differences in aptamer binding profiles across cellular models have been previously reported and may reflect differences in cellular context and target accessibility [20]. Thus, the reduced binding observed in PC3 may not be exclusive to Wy-5a and could potentially reflect characteristics of the PC3 cellular context, including differences in the abundance or accessibility of cell-surface molecules affected by trypsin treatment. However, this possibility was not directly investigated in the present study and therefore remains to be experimentally determined. This observation highlights the context-dependent nature of aptamer binding and further emphasizes the importance of validating candidate aptamers across different prostate cancer models.

Validation in patient-derived tissues further strengthens the translational relevance of the selected aptamers. While established prostate cancer cell lines remain indispensable experimental models, they cannot fully reproduce the cellular architecture and molecular complexity of human tumors. Tissue microarray analysis demonstrated preferential tumor-associated binding by all five aptamers, whereas only minimal fluorescence was observed in non-neoplastic tissues. The preservation of aptamer recognition in patient-derived tissues indicates that the recognized targets remain accessible within clinically relevant specimens despite the increased complexity of the tumor microenvironment. Although these findings are encouraging, the present analysis was performed using a single tissue microarray with distinct clinical samples, and larger independent cohorts will be necessary to determine the sensitivity, specificity, and diagnostic performance of these aptamers across the full spectrum of prostate cancer pathology.

Beyond their tumor-selective recognition, the aptamers also exhibited functional effects by reducing both cell metabolic activity and proliferation. All five aptamers significantly decreased cell metabolic activity after 48 h of treatment, whereas no significant effects were observed after 24 h. Furthermore, unlike untreated cultures, most aptamer-treated cells failed to exhibit the progressive increase in metabolic activity expected over time, suggesting that the aptamers may interfere with cellular growth dynamics rather than inducing an immediate cytotoxic response. This interpretation was further supported by the CyQUANT assays, in which all five aptamers also reduced cell proliferation after 48 h, with the most pronounced effects observed in PC3 cells.

Notably, the strongest antiproliferative effects observed in PC3 cells occurred despite the absence of tumor-selective binding relative to RWPE-1, suggesting that the magnitude of aptamer binding does not necessarily predict the magnitude of the biological response. This apparent discrepancy may reflect differences in the molecular context of the cell lines. Aptamer binding may depend, at least in part, on the abundance and accessibility of its molecular target; however, the observed functional effects may also be influenced by differences in downstream cellular pathways. Thus, PC3 cells may exhibit lower expression or accessibility of the putative aptamer target, resulting in comparatively lower binding, while simultaneously displaying higher expression or activity of proteins involved in proliferative, survival, or cell-cycle pathways. In this context, even a relatively lower level of target engagement could potentially result in a stronger downstream functional response. Conversely, higher target expression and stronger binding in another cell line may not necessarily translate into a proportionally greater biological effect if downstream signaling is less active or differently regulated.

Because the molecular targets of the aptamers have not yet been identified, these mechanisms remain hypothetical and cannot be established from the present data. Nevertheless, the observed differences between binding and functional responses indicate that binding intensity and biological activity should be considered complementary but distinct parameters during aptamer characterization.

Some limitations of the present study should be acknowledged. The molecular targets recognized by the selected aptamers have not yet been identified, precluding a mechanistic understanding of their binding profiles and biological effects. In addition, tissue validation was performed using a single commercial tissue microarray comprising a limited number of specimens (including metastatic adenocarcinoma), and larger independent cohorts will be required to further evaluate the potential diagnostic relevance of these aptamers. A further limitation is that scrambled or random-sequence oligonucleotide controls were not included in the metabolic activity and proliferation assays. Although the random-sequence aptamer library showed minimal binding to the evaluated prostate cancer cells in the flow-cytometry experiments, this finding does not fully exclude nonspecific effects of oligonucleotide exposure on cellular metabolic activity or proliferation. Finally, the biological effects reported here were evaluated exclusively in vitro, and their therapeutic relevance remains to be confirmed using more physiologically relevant experimental models. Future studies should therefore include more complex in vitro systems, such as spheroids, organoids, and 3D bioprinted tumor models, which may better recapitulate the cellular interactions, three-dimensional architecture, and microenvironmental features of prostate tumor. These models could provide an intermediate step toward the evaluation of aptamer activity under more physiologically relevant conditions before advancing to appropriate in vivo models. Addressing these limitations will be essential for further validating the therapeutic potential of these candidates and advancing them toward clinical translation.

Although differences in recognition profiles were observed among the five aptamers, the complementary performance exhibited by most candidates across the cellular and tissue-based assays suggests that a panel of aptamers with distinct binding characteristics may represent a more effective strategy for addressing the molecular heterogeneity of prostate cancer than relying on a single ligand.

## 5. Conclusions

This study characterized five DNA aptamers previously selected by Cell-SELEX against prostate cancer cells. The selected aptamers demonstrated distinct predicted structural characteristics, selective recognition of prostate cancer cells, binding to patient-derived tissues, and biological activity by reducing cell metabolic activity and proliferation in 2D cell culture models.

Overall, these findings support the potential of these aptamers as candidate molecules for further investigation in prostate cancer research. Additional studies using more physiologically relevant models and subsequent in vivo investigations will be necessary to determine their potential applicability in diagnostic, imaging, or therapeutic approaches. The complementary properties observed among the aptamers may also support the future investigation of multi-aptamer strategies to address the heterogeneity of prostate cancer.

## Figures and Tables

**Figure 1:**
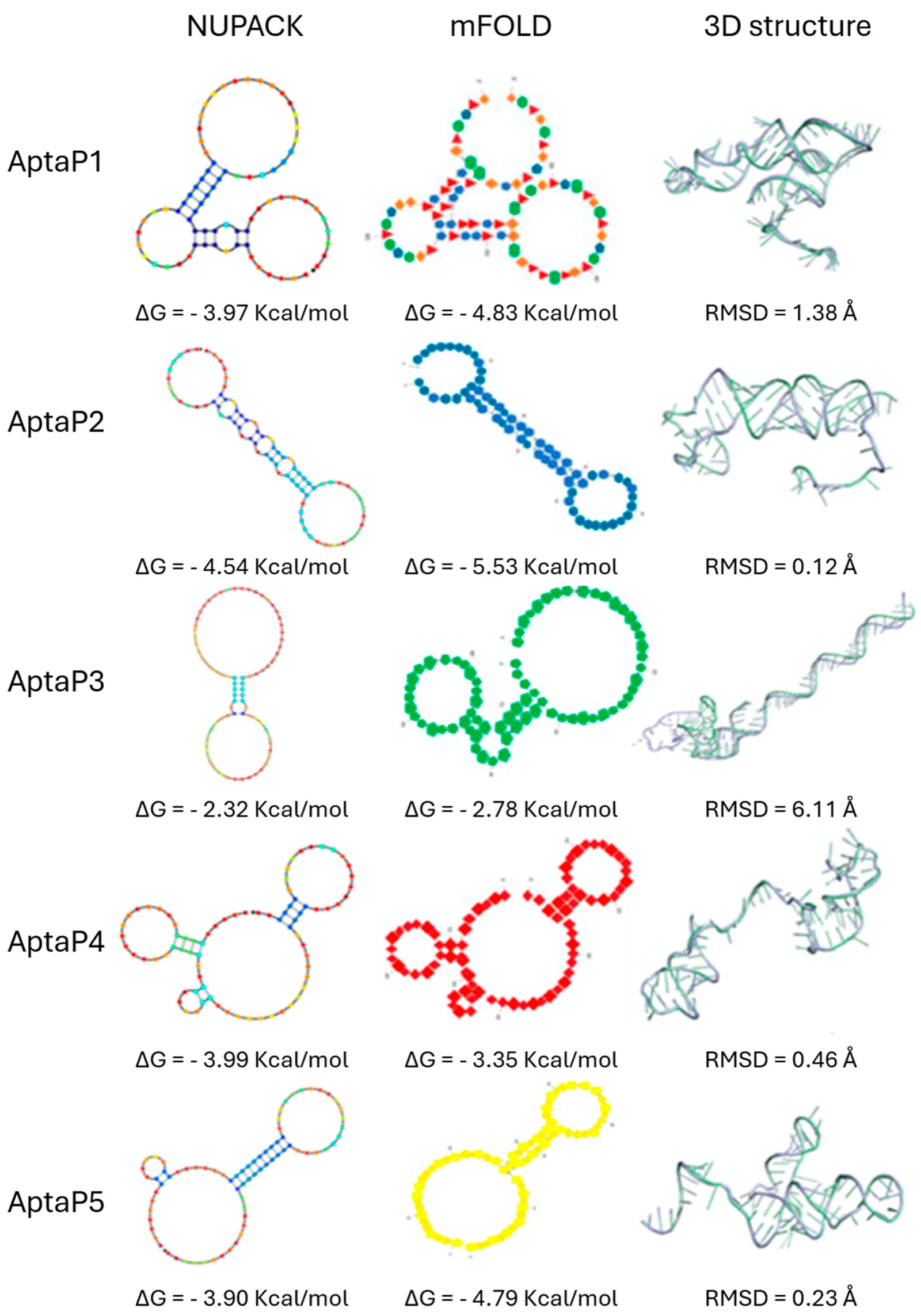
Representation of the secondary and tertiary structure predictions of the aptamers selected for the LNCaP cells (AptaP1-AptaP5). The first column shows the results obtained with the NUPACK server and the second column shows the data from the mFold server. Below the structures, the free energy ΔG values are presented. The third column shows the Tertiary structure visualizations generated using PyMOL.

**Figure 2:**
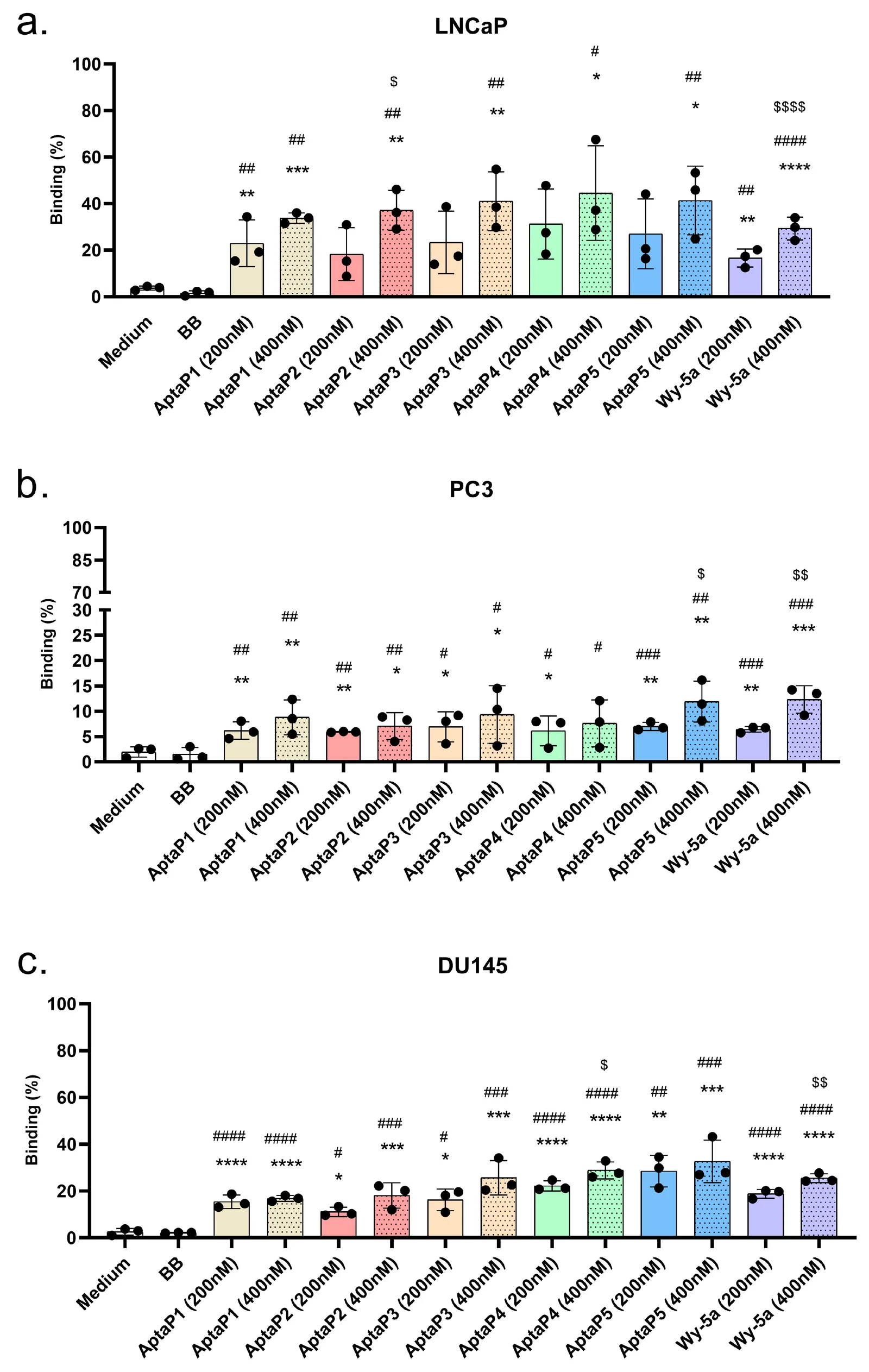
Flow cytometric analysis of AptaP1–AptaP5 and Wy-5a binding to prostate cancer cell lines. FAM-conjugated aptamers were incubated with (**a**) LNCaP, (**b**) PC3, and (**c**) DU145 cells. Medium and library-binding buffer (BB) were included as negative controls. *p* < 0.05 (*, #, $), *p* < 0.01 (**, ##, $$), *p* < 0.001 (***, ###), *p* < 0.0001 (****, ####, $$$$). * indicates comparisons with the medium control; # with BB; and $ between 200 nM and 400 nM. N = 3 independent experiments.

**Figure 3:**
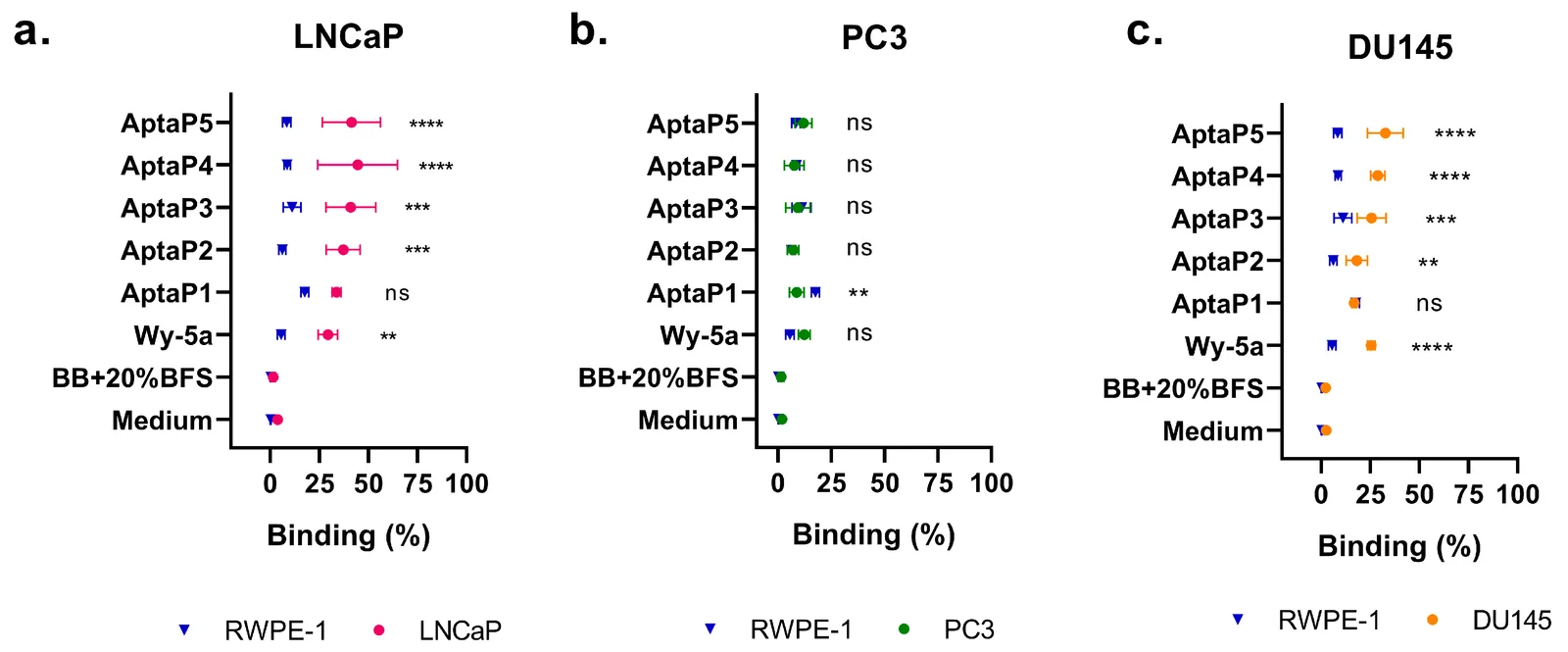
Flow cytometric analysis of the tumor cell selectivity of AptaP1–AptaP5 and Wy-5a. Binding of FAM-conjugated aptamers to (**a**) LNCaP, (**b**) PC3, and (**c**) DU145 cells was compared with binding to the non-tumorigenic prostate epithelial cell line RWPE-1. For each treatment group, binding in the tumor cell line was compared with the corresponding treatment group in RWPE-1. *p* < 0.01 (**), *p* < 0.001 (***), *p* < 0.0001 (****), ns = not significant. N = 3 independent experiments.

**Figure 4:**
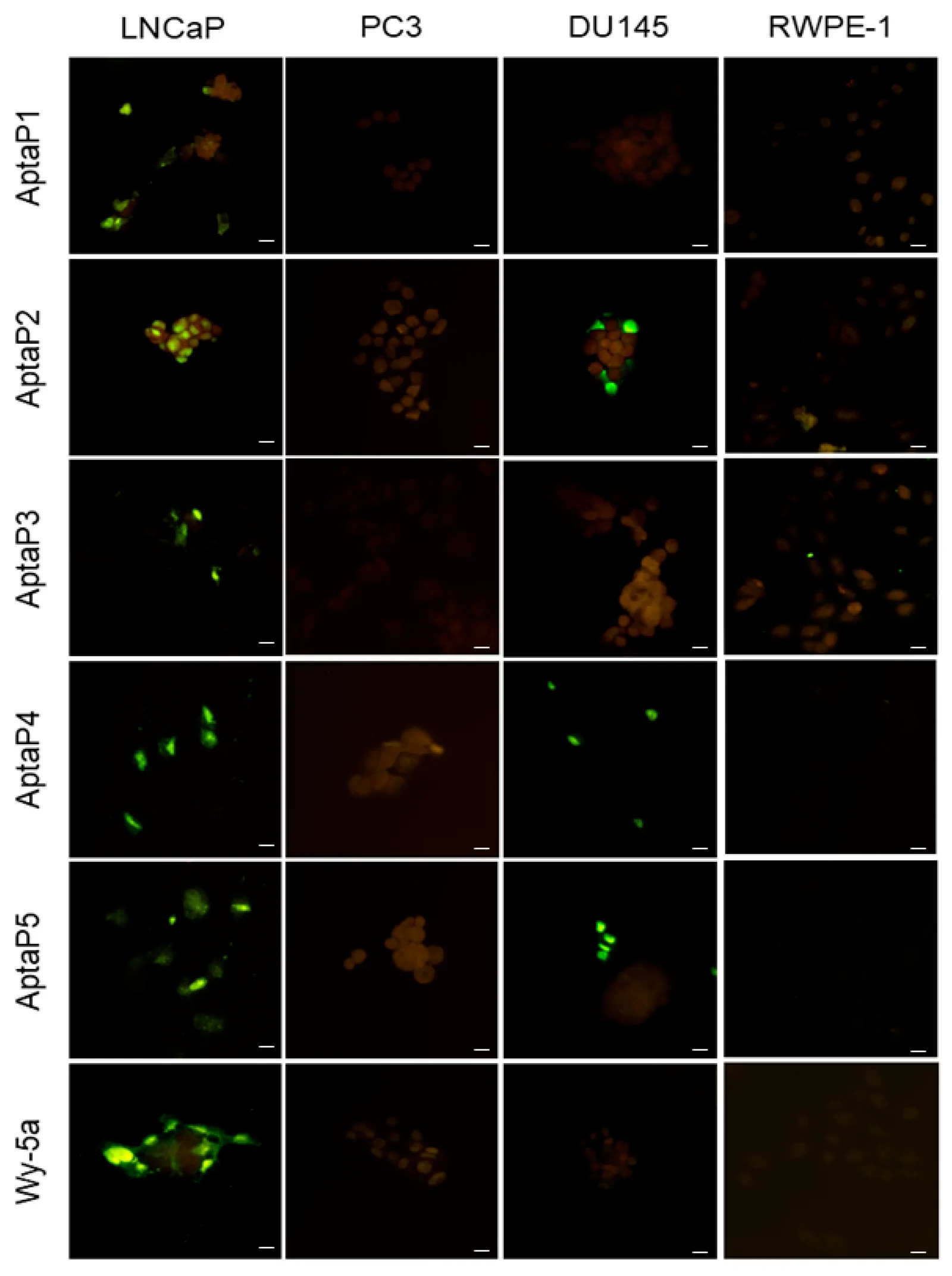
Qualitative aptafluorescence images of prostate cell lines. Representative fluorescence microscopy images showing the FAM signal (green) following incubation of LNCaP, DU145, PC3, and RWPE-1 cells with FAM-conjugated AptaP1–AptaP5 and Wy-5a. Images were acquired using a Nikon Eclipse microscope equipped with a 20× objective. N = 3 independent experiments.

**Figure 5:**
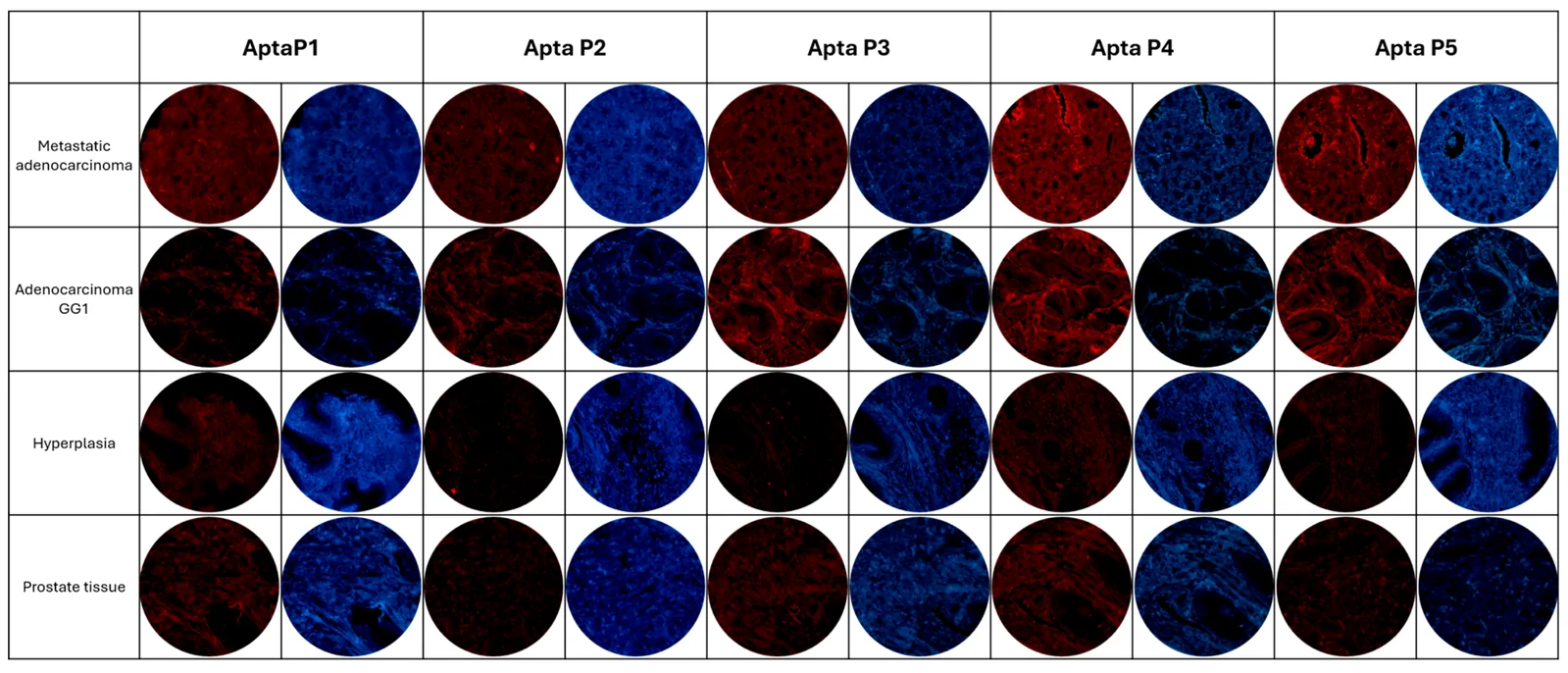
Representative images of aptamer binding to prostate tissue in a tissue microarray (TMA). Fluorescence microscopy images of prostate tissue specimens incubated with Cy5-conjugated AptaP1–AptaP5. For each aptamer, images acquired in the Cy5 and DAPI channels are shown. Cy5 fluorescence (red) indicates aptamer binding, while DAPI fluorescence (blue) indicates nuclear staining. Representative tissue specimens from metastatic adenocarcinoma, Grade Group 1 (GG1) adenocarcinoma, hyperplasia, and prostate tissue are shown. All images were acquired under the same experimental and imaging conditions.

**Figure 6:**
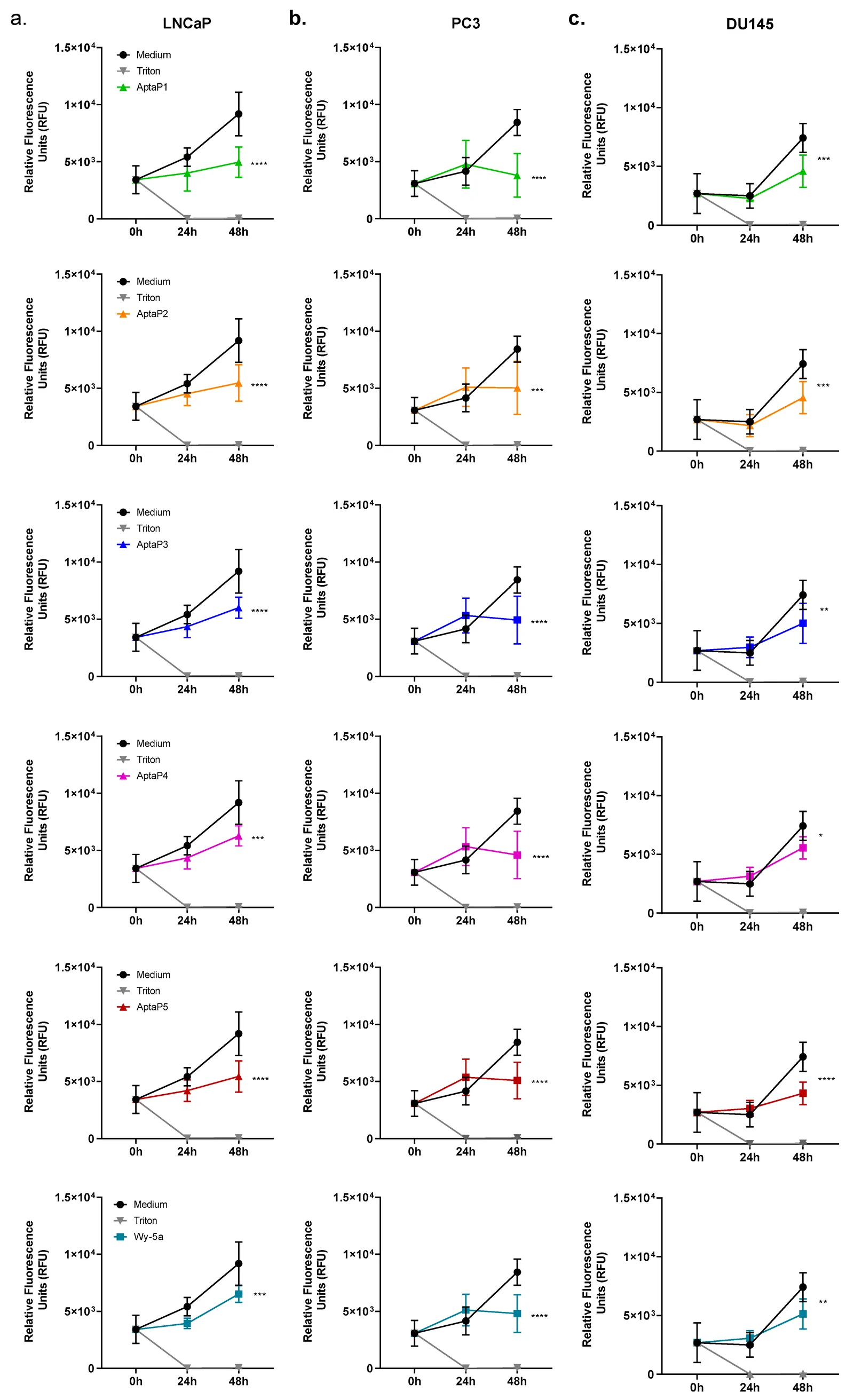
Metabolic activity in (**a**) LNCaP, (**b**) PC3, and (**c**) DU145 cells following treatment with aptamers. Relative fluorescence units (RFU) were measured at 0, 24, and 48 h after treatment. Medium and Triton X-100 (0.01%) were used as negative and positive controls, respectively. *p* < 0.05 (*), *p* < 0.01 (**), *p* < 0.001 (***), *p* < 0.0001 (****). N = 3 independent experiments.

**Figure 7:**
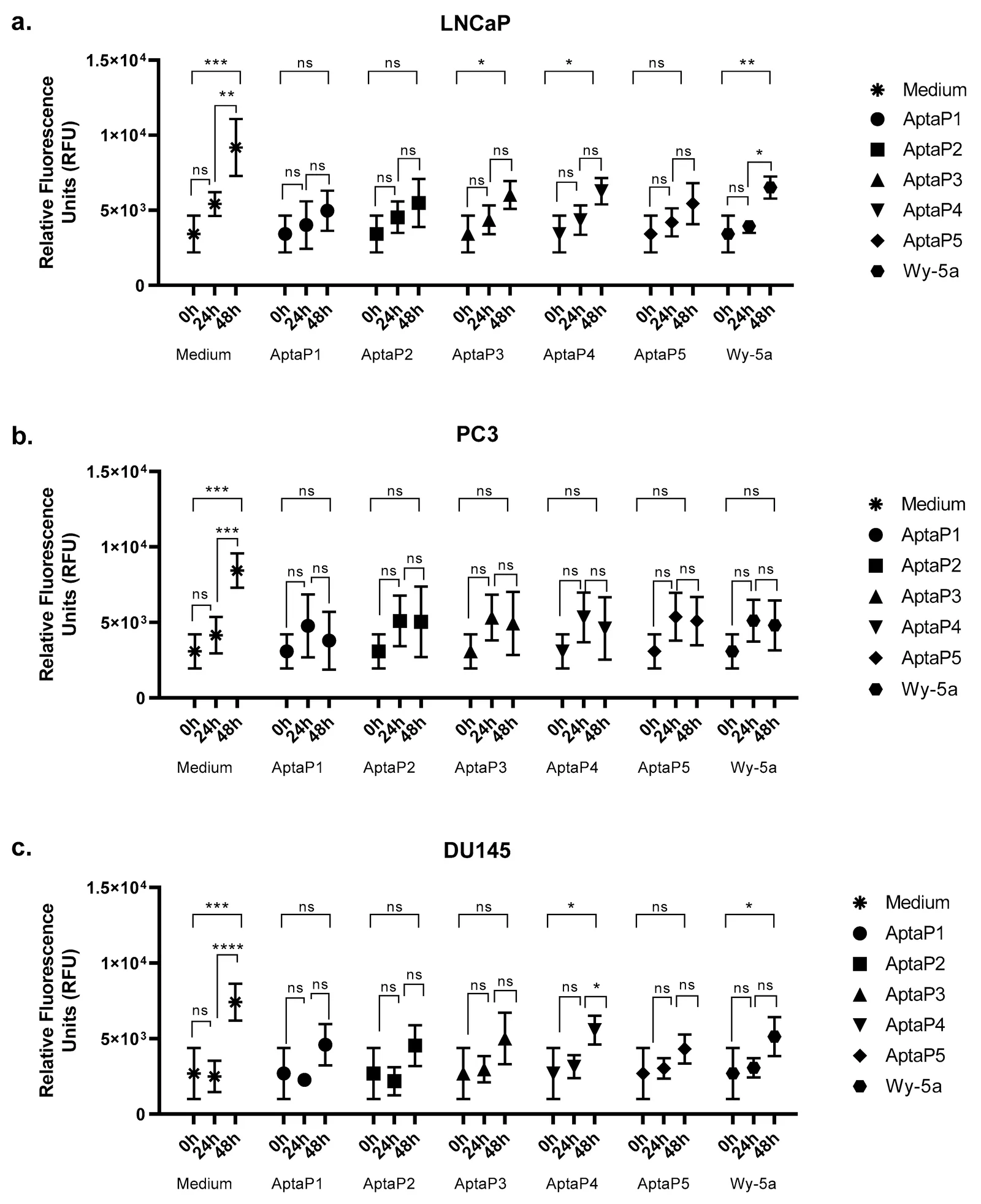
Evaluation of metabolic activity following aptamer treatment at 24 and 48 h. Metabolic activity was assessed at 24 and 48 h after treatment in (**a**) LNCaP, (**b**) PC3, and (**c**) DU145 cells. *p* < 0.05 (*), *p* < 0.01 (**), *p* < 0.001 (***), *p* < 0.0001 (****), ns = not significant. N = 3 independent experiments.

**Figure 8:**
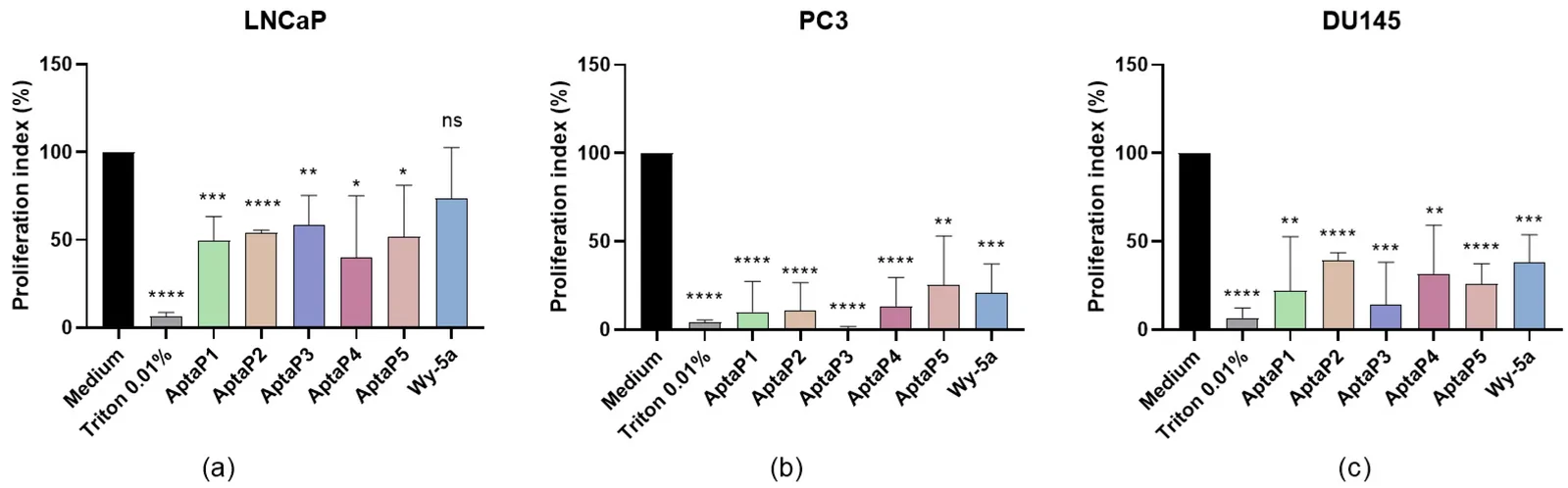
Evaluation of cell proliferation following aptamer treatment. Cell proliferation was assessed after 48 h of treatment with the five selected DNA aptamers (AptaP1–AptaP5) in (**a**) LNCaP, (**b**) PC3, and (**c**) DU145 prostate cancer cells. *p* < 0.05 (*), *p* < 0.01 (**), *p* < 0.001 (***), *p* < 0.0001 (****), ns = not significant. N = 3 independent experiments.

## Data Availability

The original contributions presented in this study are included in the article/Supplementary Materials. Further inquiries can be directed to the corresponding author.

## Supplementary Materials

The following supporting information can be downloaded at https://www.mdpi.com/article/10.3390/biom16101412/s1, Figure S1: Binding assay of the selected aptamers in the LNCaP tumor cell line by flow cytometry; Figure S2: Binding assay of the selected aptamers in the PC3 tumor cell line by flow cytometry; Figure S3: Binding assay of the selected aptamers in the DU145 tumor cell line by flow cytometry; Figure S4: Binding assay of the selected aptamers in the RWPE-1 cell line by flow cytometry.

## Abbreviations

The following abbreviations are used in this manuscript:

3D	Tridimensional
BCRJ	Rio de Janeiro Cell Bank
BPE	Bovine Pituitary Extract
BSA	Bovine Serum Albumin
CO_2_	Carbon Dioxide
DABCO	1,4-Diazabicyclo[2.2.2]Octane
DAPI	4′,6-Diamidino-2-Phenylindole
DNA	Deoxyribonucleic Acid
DPBS	Dulbecco’s Phosphate-Buffered Saline
EDTA	Ethylenediaminetetraacetic Acid
FAM	Carboxyfluorescein
GG1	Grade Group 1
HEPES	4-(2-Hydroxyethyl)-1-Piperazineethanesulfonic Acid
MgCl_2_	Magnesium Chloride
PDB	Protein Data Bank
PC	Prostate Cancer
PSA	Prostate-Specific Antigen
PSMA	Prostate-Specific Membrane Antigen
rhEGF	Recombinant Human Epidermal Growth Factor
RNA	Ribonucleic Acid
RPMI	Roswell Park Memorial Institute
ssDNA	Single-Stranded DNA
TBS	Tris-Buffered Saline
TMA	Tissue Microarray
ΔG	Gibbs Free Energy Change
