# Supplementary material for: Aptamers as Innovative Tools for Prostate Cancer: Tumor Recognition and Biological Activity
Source: Biomolecules. 2026 Sep 29;16(10):1412. doi: 10.3390/biom16101412 (PMC13702447; doi:10.3390/biom16101412)
Supplement: Supplementary file 1 [file biomolecules-16-01412-s001.zip › Figure S1.pdf]

LNCaP

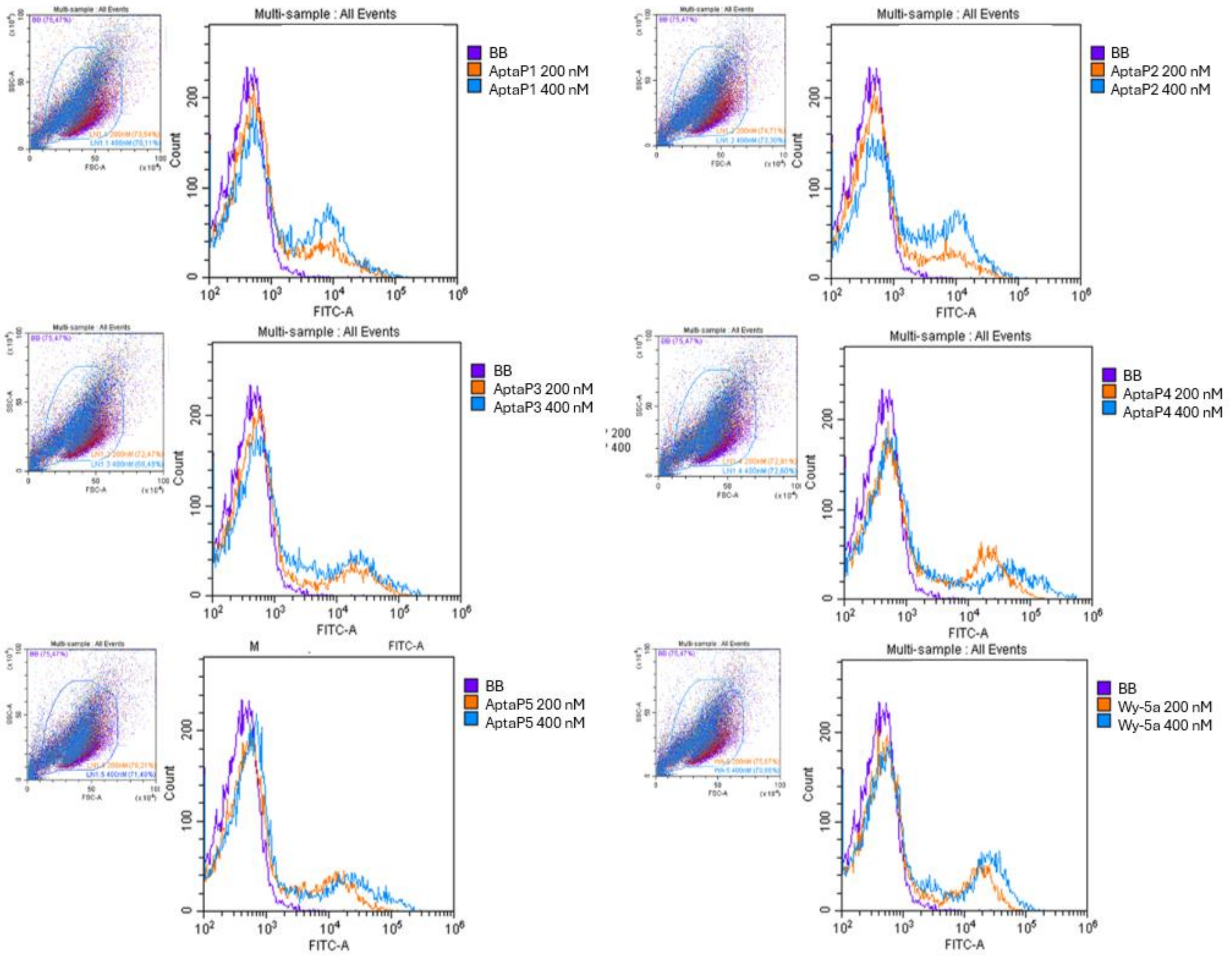
Multi-sample : All Events
BB
AptaP1 200 nM
AptaP1 400 nM
Count
FITC-A
Multi-sample : All Events
BB
AptaP2 200 nM
AptaP2 400 nM
Count
FITC-A
Multi-sample : All Events
BB
AptaP3 200 nM
AptaP3 400 nM
Count
FITC-A
Multi-sample : All Events
BB
AptaP4 200 nM
AptaP4 400 nM
Count
FITC-A
M
FITC-A
BB
AptaP5 200 nM
AptaP5 400 nM
Count
FITC-A
Multi-sample : All Events
BB
Wy-5a 200 nM
Wy-5a 400 nM
Count
FITC-A
