# Supplementary material for: Aptamers as Innovative Tools for Prostate Cancer: Tumor Recognition and Biological Activity
Source: Biomolecules. 2026 Sep 29;16(10):1412. doi: 10.3390/biom16101412 (PMC13702447; doi:10.3390/biom16101412)
Supplement: Supplementary file 1 [file biomolecules-16-01412-s001.zip › Supplementary Figures Legends.pdf]

Supplementary Figures Legends

Supplementary Figure S1:

Binding assay of the selected aptamers in the LNCaP tumor cell line by flow cytometry. Representative histograms of the flow cytometry binding assay of FAM-labeled aptamers AptaP1, AptaP2, AptaP3, AptaP4, AptaP5, and Wy-5a at concentrations of 200 nM (orange) and 400 nM (blue), with Binding Buffer as the negative control (purple). Histograms illustrate the overlay of each aptamer at the two concentrations and its corresponding Binding Buffer negative control (red). *n* = 3.

Supplementary Figure S2:

Binding assay of the selected aptamers in the PC3 tumor cell line by flow cytometry. Representative histograms of the flow cytometry binding assay of FAM-labeled aptamers AptaP1, AptaP2, AptaP3, AptaP4, AptaP5, and Wy-5a at concentrations of 200 nM (orange) and 400 nM (blue), with Binding Buffer as the negative control (purple). Histograms illustrate the overlay of each aptamer at the two concentrations and its corresponding Binding Buffer negative control (red). *n* = 3.

Supplementary Figure S3:

Binding assay of the selected aptamers in the DU145 tumor cell line by flow cytometry. Representative histograms of the flow cytometry binding assay of FAM-labeled aptamers AptaP1, AptaP2, AptaP3, AptaP4, AptaP5, and Wy-5a at concentrations of 200 nM (orange) and 400 nM (blue), with Binding Buffer as the negative control (purple). Histograms illustrate the overlay of each aptamer at the two concentrations and its corresponding Binding Buffer negative control (red). *n* = 3.

Supplementary Figure S4:

Binding assay of the selected aptamers in the RWPE-1 cell line by flow cytometry. Representative histograms of the flow cytometry binding assay of FAM-labeled aptamers AptaP1, AptaP2, AptaP3, AptaP4, AptaP5, and Wy-5a at concentrations of 200 nM (orange) and 400 nM (blue), with Binding Buffer as the negative control (purple). Histograms illustrate the overlay of each aptamer at the two concentrations and its corresponding Binding Buffer negative control (red). *n* = 3.
